# Superoxide Dismutase Gene Family in Chili Pepper (*Capsicum annuum* L.): Molecular Characterization and Involvement in Redox Regulation Under Chilling Stress

**DOI:** 10.3390/antiox14091131

**Published:** 2025-09-18

**Authors:** Seo Hyeon Ban, Chae Eun Song, Seung Hee Eom, Tae Kyung Hyun

**Affiliations:** Department of Industrial Plant Science and Technology, College of Agriculture, Life and Environment Sciences, Chungbuk National University, Cheongju 28644, Republic of Korea

**Keywords:** antioxidant, chili pepper, *Capsicum annuum*, chilling stress, superoxide dismutase

## Abstract

Chilling stress is a major abiotic factor that limits chili pepper (*Capsicum annuum* L.) cultivation by disrupting redox homeostasis, thereby impairing growth and fruit productivity. Superoxide dismutases (SODs), which catalyze the conversion of superoxide radicals into hydrogen peroxide and oxygen, serve as key components of the plant antioxidant defense system. However, the SOD gene family in chili pepper has not been comprehensively characterized. Therefore, this study aimed to characterize the SOD gene family in chili pepper and investigate their responses to chilling stress. We identified nine putative *CaSOD* genes and classified them into CZSOD, FeSOD, and MnSOD clades based on phylogenetic relationships and conserved domain architecture. Bioinformatic analyses revealed variation in physicochemical properties and predicted subcellular localizations, suggesting functional diversification. Transcriptome profiling indicated tissue-specific expression, with several *CaSODs* preferentially expressed in fruits and floral buds, while qRT-PCR analysis demonstrated that six *CaSODs* were transcriptionally induced under chilling stress. Functional validation in *Nicotiana benthamiana* leaves showed that transient expression of four selected *CaSODs* significantly enhanced SOD activity in an isoform-specific manner. Future studies should validate these genes across diverse chili pepper cultivars under field conditions and assess their potential for integration into breeding programs. Collectively, these findings provide new insights into the molecular and functional diversity of CaSODs, highlight their role in maintaining redox balance under chilling stress, and provide useful genetic resources for breeding stress-tolerant chili pepper and related crops.

## 1. Introduction

Chili pepper (*Capsicum annuum* L.) is a globally cultivated crop of high economic value, prized for its culinary uses and rich content of bioactive compounds such as capsaicinoids, ascorbic acid, and carotenoids [1,2]. Despite its broad adaptability, chili pepper is inherently sensitive to chilling stress due to its subtropical origin. Chilling stress, generally defined as exposure to non-freezing temperatures below 15 °C, disrupts cellular homeostasis and impairs key physiological processes essential for seedling establishment and fruit development. In regions such as China, South Korea, and Turkey, chili pepper seedlings are frequently exposed to low temperatures, often below 10 °C, from late night to early morning during late spring, leading to various chilling-induced symptoms [3,4,5]. These symptoms include stem flaccidity, foliar wilting, tissue necrosis, and increased susceptibility to opportunistic pathogens, all of which severely compromise plant growth and survival [3,6]. Consequently, chilling stress represents a major abiotic constraint that hampers early development, reduces yield stability, and diminishes the economic returns for chili pepper producers.

Exposure to chilling stress causes damage to cellular membranes, including thylakoid membranes, primarily due to lipid destabilization, protein denaturation, and solute leakage induced by excessive accumulation of reactive oxygen species (ROS) [7,8]. The oxidative injury and disruption of cellular homeostasis caused by ROS are mitigated by a complex antioxidant defense system. This system comprises enzymatic antioxidants, such as catalase, superoxide dismutase (SOD), peroxidase, glutathione reductase, and glutathione peroxidase, as well as non-enzymatic components like ascorbic acid, carotenoids, α-tocopherol, and glutathione [8]. Within this group, SODs, a widely conserved family of metalloenzymes, act as the primary defense against oxidative stress by catalyzing the conversion of superoxide radicals into hydrogen peroxide (H_2_O_2_) and molecular oxygen (O_2_) [9]. In plants, SOD activity is typically upregulated under cold stress, contributing to enhanced tolerance by mitigating oxidative damage associated with chilling stress. For example, overexpression of *AtSOD* (*Arabidopsis thaliana SOD*) and *CmSOD* (*Cucurbita moschata SOD*) in Arabidopsis improved chilling tolerance by scavenging •O_2_^−^ [10]. Similarly, tobacco plants overexpressing *SiCSD* (*Saussurea involucrata* copper/zinc SOD) exhibited increased tolerance to drought, freezing, and oxidative stress [11]. Furthermore, transgenic Arabidopsis and yeast lines expressing *PutCZSOD* (*Puccinellia tenuiflora* copper/zinc *SOD*) showed enhanced resistance to multiple abiotic stresses [12]. Together, these findings demonstrate that enhancing *SOD* expression and activity represents a fundamental mechanism of plant adaptation to chilling stress, primarily by improving ROS detoxification.

Given their essential role in the antioxidant defense system, *SOD* genes have been extensively characterized in numerous plant species, including foxtail millet [13], garlic [9], *Platycodon grandiflorus* [14], *Daucus carota* [15], *Zostera marina* [16], members of the Rosaceae family [17], and cucurbits such as watermelon and melon [18]. However, despite the economic importance of chili pepper and its sensitivity to chilling stress, comprehensive studies on its SOD gene family remain limited. In this study, we performed a genome-wide analysis of the *SOD* gene family in chili pepper and identified nine *C. annuum SOD* (*CaSOD*) genes. Phylogenetic analysis, including homologs from diverse species, classified the *CaSOD* genes into three well-defined clades. Expression profiling across different tissues and under chilling stress revealed differential regulation among CaSOD members. Overall, this study addresses the current knowledge gap regarding SOD genes in chili pepper and provides valuable genetic resources for improving stress tolerance.

## 2. Materials and Methods

### 2.1. Plant Growth and Treatment

Chili pepper seeds (*C. annuum* cv. Nockwang), a cultivar commonly grown in South Korea, were sown and cultivated under controlled growth conditions (24 °C, 60% relative humidity, 16 h light/8 h dark photoperiod). Chilling stress was applied to 4-week-old seedlings by exposing them to 0 °C for 3, 6, or 9 h. Each treatment was conducted using three independent biological replicates.

### 2.2. In Situ H_2_O_2_ Staining and Lipid Peroxidation Measurement

Accumulation of H_2_O_2_ in chilling-stressed leaves was visualized using peroxidase-dependent 3,3′-diaminobenzidine (DAB) staining, following the method of Ji et al. [9]. Briefly, leaves were vacuum-infiltrated with DAB solution, incubated in darkness for 12 h, and subsequently decolorized with 70% ethanol to remove chlorophyll.

Chilling-induced oxidative damage was further assessed by quantifying lipid peroxidation through malondialdehyde (MDA) content determined using the thiobarbituric acid reaction as described by Ji et al. [9]. About 100 mg of ground material obtained from each sample was used. MDA concentration was estimated by subtracting the non-specific turbidity at 600 nm from the absorption at 532 nm. MDA levels were expressed relative to the mock control.

### 2.3. In-Gel SOD Activity Assay and Total SOD Activity Analysis

Total proteins were extracted using a buffer containing 0.2 M potassium phosphate (pH 7.8), 0.1 mM EDTA, and 1% protease inhibitor cocktail, followed by centrifugation at 14,000× *g* for 15 min at 4 °C. Supernatants were collected, and protein concentrations were determined using the BCA assay (Pierce™, Thermo Fisher Scientific, Waltham, MA, USA).

For native SOD activity profiling, proteins were resolved on 10% native-PAGE and stained with riboflavin-NBT solution [19] under dark conditions. Gels were then exposed to light to visualize SOD activity as achromatic bands. Isoforms were distinguished by pre-incubating gels with either 1 mM KCN or 5 mM H_2_O_2_.

Total SOD activity was quantified according to the protocol described by Elavarthi and Martin [19].

### 2.4. In Silico Identification and Phylogenetic Analysis of CaSOD Family

Members of the CaSOD gene family were identified through a genome-wide BLAST (SequenceServer version 2.2.0) search using A. thaliana SOD proteins as queries. The putative CaSOD genes were subjected to comprehensive bioinformatic characterization, including analyses of conserved domains, molecular weight, isoelectric point (pI), and predicted subcellular localization as described by Hyun [14]. Phylogenetic analysis classified the CaSOD proteins into distinct subgroups, providing insights into their evolutionary diversification.

### 2.5. Gene Expression Analysis

Tissue-specific expression profiles of CaSOD genes, including root, stem, leaves, buds, flowers, and nine developmental stages of fruit, were examined using RNA-Seq datasets [20]. Gene expression levels were represented as Z-scores derived from FPKM values.

Total RNA was extracted from chilling-treated leaves and used for cDNA synthesis via reverse transcription. Quantitative real-time PCR (qRT-PCR) was performed according to the protocol of Ahn et al. [21], and expression levels were normalized against the constitutively expressed CaUBI3 gene (GenBank accession no. AY486137.1). Relative gene expression levels were calculated using the 2^−ΔΔCt^ method as implemented in the Bio-Rad CFX Manager Software (Version 3.1) (Bio-Rad, Hercules, CA, USA). Primer sequences are listed in Table S1.

### 2.6. Cloning and Transient Expression of CaSODs in Nicotiana Benthamiana

Full-length *CaSOD* coding sequences were amplified using gene-specific primers (Table S1), cloned into the pENTR/D-TOPO entry vector, and subsequently sub-cloned into the pGWB505 binary vector. Transient expression in *N. benthamiana* leaves was performed as previously described [22]. Agrobacterium tumefaciens strain GV3101 harboring each *CaSOD* construct was infiltrated into tobacco leaves using a needleless syringe. Leaf tissues were harvested three days post-infiltration for SOD activity analysis. All experiments were conducted with three independent biological replicates.

### 2.7. Statistical Analysis

Results are shown as mean ± SD from at least three biological replicates. Statistical differences were assessed by one-way ANOVA with Duncan’s multiple range test (*p* < 0.05) using SPSS v25 (IBM Corp., Armonk, NY, USA).

## 3. Results and Discussion

### 3.1. Physiological and Biochemical Responses of Chili Pepper Plants to Chilling Stress

**Figure 1:**
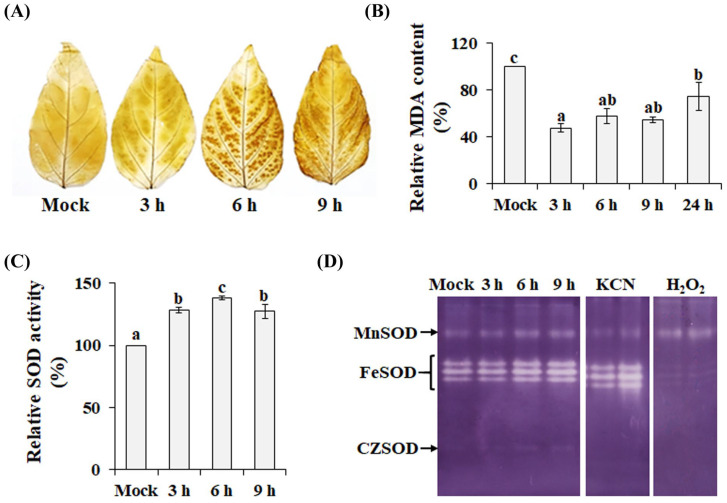
Physiological responses of chili pepper plants to chilling stress. Levels of H_2_O_2_ (**A**), malondialdehyde (MDA) (**B**), total SOD activity (**C**), and SOD isozyme activity patterns (**D**) were analyzed. Mean values ± SD were obtained from three independent replicates. Groups marked with different letters differ significantly (*p* < 0.05).

Chilling is a major stress factor that disrupts the balance between ROS production and the antioxidant defense system. To evaluate the impact of chilling, physiological responses of chili pepper plants were examined. As shown in Figure 1A, chilling treatment caused notable H_2_O_2_ accumulation in chili pepper leaves, as indicated by the dark brown coloration observed after DAB staining. In contrast, MDA, a stable end product of lipid peroxidation [23], exhibited a biphasic response: compared with the mock control, chilling treatment caused a significant reduction in MDA content. From 3 to 9 h of chilling exposure, the MDA level remained relatively stable without significant changes; however, a marked increase was observed when the stress persisted beyond 24 h (Figure 1B). Similar patterns have been reported in other plant species exposed to cold stress. For instance, in oil palm (*Elaeis guineensis*), MDA levels decreased during the first 24 h of chilling but increased thereafter as oxidative damage accumulated [24]. Moreover, cold stress has been shown to induce MDA accumulation within 1 to 3 days of treatment in a variety of plant species [25,26,27]. These observations confirm that the chilling treatment was physiologically effective. To further assess the response, leaves from plants grown under both control and chilling conditions were analyzed for SOD activity. Compared with the mock control (plants maintained under non-stress conditions for 9 h), chilling stress induced a significant increase in total SOD activity (Figure 1C).

Based on their metal cofactors, plant SODs are generally classified into three major types: copper/zinc SOD (CZSOD), iron SOD (FeSOD), and manganese SOD (MnSOD) [14]. To investigate isozyme-specific responses, CaSOD isoforms were separated by native PAGE and treated with selective inhibitors—KCN (CZSOD inhibitor) and H_2_O_2_ (inhibitor of both FeSOD and CZSOD). As shown in Figure 1D, the activities of all SOD isoforms were consistently higher in mock control plants than in chilling-stressed plants. Among them, CaFeSODs displayed the highest activity and remained constitutively expressed under both control and stress conditions. FeSODs are known to maintain high activity across diverse plant species [9,28,29], suggesting their central role in both baseline and stress-induced ROS detoxification.

### 3.2. Identification and Characterization of CaSODs

To identify SOD gene candidates in chili pepper, a homology-based search was performed using functionally validated SOD protein sequences from *A. thaliana* as reference queries, as described in the materials and methods. Sequence similarity searches against the chili pepper genome database (Zunla-1_v3.0.genome, PRJCA025503) using BLASTP resulted in the identification of nine putative *CaSOD* genes (Table 1). To further characterize these proteins, their physicochemical properties—including predicted molecular weight, pI, and subcellular localization—were analyzed using the ExPASy and WoLF PSORT tools. The CaSOD proteins ranged in length from 157 amino acids (CaCZSOD3) to 313 amino acids (CaCZSOD2), with corresponding molecular weights of 15.87 to 34.23 kDa and theoretical pIs spanning from 5.94 to 9.73, reflecting substantial structural diversity.

**Figure 2:**
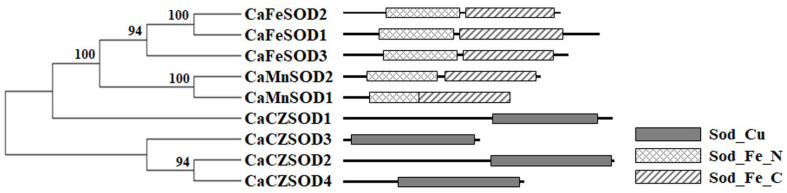
Genome-wide analysis of the *SOD* gene family in chili pepper. Phylogenetic relationships among CaSOD proteins were constructed using the neighbor-joining method in MEGA7. Conserved domain structures of CaSODs identified using SMART analysis.

To elucidate evolutionary relationships among the CaSOD proteins, a phylogenetic tree was reconstructed using the neighbor-joining method (Figure 2 and Figure S1). This analysis resolved the CaSOD proteins into three principal clades, corresponding to distinct structural domains and phylogenetic patterns (Figure 2). Domain analysis revealed the presence of the conserved Sod_Cu domain (PF00080) in CaCZSOD1 to CaCZSOD4, supporting their classification as CZSODs. In addition, the chili pepper genome encodes three FeSOD proteins and two MnSODs, each containing the characteristic iron/manganese superoxide dismutase N-terminal (Sod_Fe_N, PF00081) and C-terminal (Sod_Fe_C, PF02777) domains (Figure 2). In higher plants, CZSODs are distributed across multiple subcellular compartments, whereas FeSODs and MnSODs are primarily localized to chloroplasts and mitochondria, respectively [9,14]. Consistent with this pattern, CaCZSODs were predicted to localize to either the chloroplast or cytoplasm, while CaFeSODs and CaMnSODs were predicted to reside in the chloroplast and mitochondria, respectively (Table 1). This distribution suggests that CaSOD isoforms function cooperatively across subcellular compartments to maintain redox balance within the cell.

### 3.3. Expression Patterns of CaSODs Across Different Tissues and in Response to Chilling Stress

**Figure 3:**
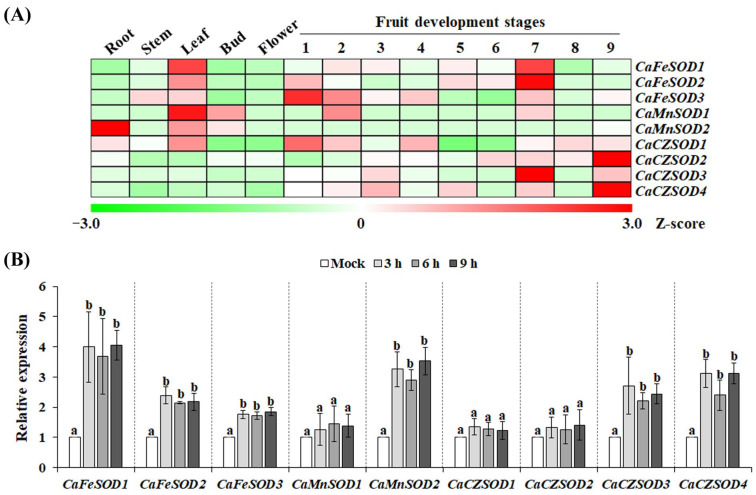
Expression profiles of the *SOD* gene family in chili pepper. (**A**) Tissue-specific expression of *CaSOD* genes in roots, stems, leaves, buds, flowers, and nine developmental stages of fruit. Expression levels are shown as Z-scores of FPKM values from RNA-Seq data. (**B**) Expression of *CaSOD* genes under chilling stress. Transcript levels were normalized to *CaUBI3* and presented relative to the mock control. Data in bars are expressed as mean ± SD (*n* = 3). Groups labeled with different letters differ significantly at *p* < 0.05.

Tissue-specific transcriptional analysis provides valuable insights into whether a target gene contributes to the functional differentiation of specific tissues. To evaluate the expression profile of *CaSODs*, transcript levels were analyzed using RNA-Seq datasets derived from different tissues. As shown in Figure 3A, *CaFeSODs* were highly expressed in leaves and fruits, whereas *CaMnSODs* showed high transcript levels in leaves and buds. In apple, SOD activity during early bud swelling has been shown to play a crucial role in dormancy release by scavenging free radicals [30]. This suggests that *CaMnSODs* may similarly contribute to bud reactivation in chili pepper by alleviating oxidative stress. Notably, most *CaSOD* genes—except *CaMnSOD2*—displayed elevated expression in fruits. In guava (*Psidium guajava* L.) fruits, SOD activity increased during ripening but declined thereafter, leading to reduced antioxidant capacity and ROS accumulation, which in turn caused tissue degradation during over-ripening [31]. These findings imply that fruit-expressed *CaSOD* genes may be critical for maintaining redox homeostasis during fruit development and ripening, thereby contributing to improved postharvest quality and extended shelf life of chili pepper.

In higher plants, *SOD* genes are typically induced by environmental stresses and phytohormones, although distinct expression patterns have been observed depending on the stress type [9,15,16,32,33]. To determine whether the increase in SOD activity under chilling stress corresponded with transcriptional induction of CaSODs, qRT-PCR was performed. Chilling stress triggered strong upregulation of *CaFeSOD1*, *CaFeSOD2*, *CaFeSOD3*, *CaMnSOD2*, *CaCZSOD3*, and *CaCZSOD4*. Peak transcript levels were detected 3 h after stress initiation and remained elevated up to 9 h (Figure 3B). These results indicate that the observed increase in total SOD activity (Figure 1C,D) is at least partially attributable to transcriptional activation of specific CaSODs under chilling stress (Figure 3B). Based on these expression patterns, *CaFeSOD1*, *CaMnSOD2*, *CaCZSOD3*, and *CaCZSOD4* were selected for subsequent functional analysis.

### 3.4. Molecular and Functional Characterization of Four Putative CaSODs

**Figure 4:**
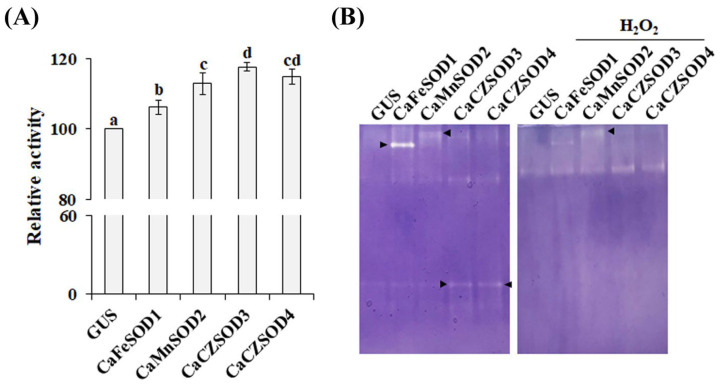
Functional validation of selected *CaSODs* in *N. benthamiana* by agroinfiltration. Total SOD activity (**A**) and isozyme activity patterns (**B**) were analyzed in leaves infiltrated with *A. tumefaciens* carrying each *CaSOD* overexpression construct or the 35S:GUS construct (mock control). Arrows indicate distinct SOD activity bands. Results are presented as mean ± SD from independent experiments, and distinct letters denote differences that are statistically significant at *p* < 0.05.

Agroinfiltration has been widely employed as a rapid and reliable system for the transient evaluation of plant gene function and protein interactions [34,35]. To investigate the functional relevance of the four selected *CaSODs*, their coding sequences were cloned under the control of the constitutive CaMV35S promoter and transiently expressed in *N. benthamiana* leaves via agroinfiltration. Three days post-infiltration, total soluble proteins were extracted and subjected to enzymatic assays to measure SOD activity. As shown in Figure 4A, infiltration with each *CaSOD* construct resulted in a substantial increase in total SOD activity compared with the mock-infiltrated control (35S:GUS). Furthermore, native gel analysis revealed distinct SOD activity bands in *CaSOD*-overexpressing leaves, exhibiting isoform-specific patterns depending on the *CaSOD* expressed (Figure 4B). Notably, leaves expressing *CaCZSOD3* or *CaCZSOD4* displayed an isoform pattern similar to that of the GUS control, although total SOD activity was significantly elevated. This observation suggests that CaCZSOD3 or CaCZSOD4 activity may overlap with endogenous CZSOD isoforms in *N. benthamiana*, thereby masking isoform-specific changes. However, as a heterologous system, *N. benthamiana* may not fully replicate in vivo responses in chili pepper, likely due to differences in post-translational protein modifications [36], and caution is required when extrapolating these findings. Nevertheless, this system provides a rapid assessment of SOD functionality and isoform specificity. Collectively, these results demonstrate that all four *CaSODs* encode functional SODs and that heterologous expression of these genes in *N. benthamiana* is sufficient to enhance overall SOD activity in leaf tissues.

## 4. Conclusions

This study presents the first comprehensive analysis of the *CaSOD* gene family in chili pepper and elucidates their role in ROS detoxification under chilling stress. By integrating bioinformatics, transcriptional profiling, and functional validation, we demonstrate that CaSOD isoforms exhibit tissue-specific functions and stress-inducible expression, thereby maintaining redox homeostasis during oxidative stress. Transient overexpression assays further confirm that *CaSODs* encode active enzymes capable of enhancing antioxidant capacity in planta. Collectively, these findings provide a systematic framework for understanding the evolutionary and functional diversification of plant SODs and identify CaSODs as promising targets for improving abiotic stress tolerance. Future research should aim to validate CaSOD functions directly in chili pepper under field conditions and across diverse cultivars. Investigating the crosstalk between CaSOD-mediated ROS detoxification and other stress-responsive pathways will provide a more comprehensive understanding of stress adaptation. Strategic manipulation of CaSODs through transgenic, genome-editing, or molecular breeding approaches could ultimately facilitate the development of chili pepper cultivars with enhanced resilience and productivity, a goal of considerable agronomic and nutritional significance.

## Figures and Tables

**Table 1:** Superoxide dismutase gene family in *Capsicum annuum*.

Name	Accession Number	Chromosome Location	pI	MW (kDa)	Localization
CaFeSOD1	ZLC03G0026460	Chr03: 101046984-101056167	6.03	34.23	Chloroplast
CaFeSOD2	ZLC06G0026510	Chr06: 171945626-171949651	8.59	28.19	Chloroplast
CaFeSOD3	ZLC12G0014730	Chr12: 128908499-128917081	7.08	29.77	Chloroplast
CaMnSOD1	ZLC01G0038490	Chr01: 273502447-273503108	9.73	21.97	Mitochondrion
CaMnSOD2	ZLC05G0019920	Chr05: 233592291-233603550	8.39	25.53	Mitochondrion
CaCZSOD1	ZLC01G0005110	Chr01: 9050068-9053210	6.54	32.81	Chloroplast
CaCZSOD2	ZLC01G0032140	Chr01: 236323717-236328354	6.07	33.67	Cytoplasm
CaCZSOD3	ZLC10G0002210	Chr10: 4241074-4245338	7.13	15.87	Cytoplasm
CaCZSOD4	ZLC11G0002710	Chr11: 5405444-5416665	5.94	21.69	Chloroplast

## Data Availability

The data presented in this study are available on request from the corresponding author. The data are not publicly available due to reasons of privacy.

## Supplementary Materials

The following supporting information can be downloaded at: https://www.mdpi.com/article/10.3390/antiox14091131/s1, Table S1: List of primers used for gene expression and functional assays. Figure S1: Phylogenetic tree of SOD proteins from Arabidopsis thaliana, rice (Oryza sativa), and chili pepper.
