# Supplementary material for: Superoxide Dismutase Gene Family in Chili Pepper (Capsicum annuum L.): Molecular Characterization and Involvement in Redox Regulation Under Chilling Stress
Source: Antioxidants (Basel). 2025 Sep 18;14(9):1131. doi: 10.3390/antiox14091131 (PMC12466808; doi:10.3390/antiox14091131)
Supplement: Supplementary file 1 [file antioxidants-14-01131-s001.zip › antioxidants-3880183-supplementary.pdf]

Table S1. List of primers used for gene expression and functional assays

	Primer	Sequences (5'-3')
For qRT-PCR	CaFeSOD1-F	ACTCAGCTGGACGAATTCAC
	CaFeSOD1-Rev	TCCACCAGGCTTCATAGACT
	CaFeSOD2-F	GCTGCTAACTCACTGTCACT
	CaFeSOD2-Rev	GTACCCGAAACAGCTTTCCT
	CaFeSOD3-F	CCTAATCGGTTTGGACGTGT
	CaFeSOD3-Rev	GGGTTCGCCTAAATTTACAAAGG
	CaMnSOD1-F	CTTCTTTCTACCGGATCTGCCG
	CaMnSOD1-Rev	TGTAGGAGCTAGGTTCCAGTTA
	CaMnSOD2-F	GACCGAACTTGGTTCCTCTT
	CaMnSOD2-Rev	GCATTCTTTCTCGTAAACTTC
	CaCZSOD1-F	TGAAGCGAACTTCAGTGGAC
	CaCZSOD1-Rev	TCGTCAACTTCCAGTGTTCC
	CaCZSOD2-F	TAGTGGCACCATCCTCTTCA
	CaCZSOD2-Rev	ACATGCAGCCATTTGTGGTA
	CaCZSOD3-F	TTGCAATTCCACTGGACCTC
	CaCZSOD3-Rev	CCATCAGGACCAGCAACAAT
	CaCZSOD4-F	TTCACGAGCTTGAGGATGAC
	CaCZSOD4-Rev	GGAGTCAAACCAACAACACC
	CaUBI3-F	GCCGACAGATAGCCCTTATG
	CaUBI3-Rev	TTAATGCTGGACTCCACTGC
For cloning	CaFeSOD1-F	CACCATGATGGCCGCCGCCACAGC
	CaFeSOD1-Rev	TTCCGCCTCAGAATCCGTAGT
	CaMnSOD2-F	CACCATGGCTCTTCGAAACCTAAT
	CaMnSOD2-Rev	AGGGCATTCTTTCTCGTAAAC
	CaCZSOD3-F	CACCATGGGAAACTTGAAAGCAGT
	CaCZSOD3-Rev	AACAGATGATTGAAGCCCAAT
	CaCZSOD4-F	CACCATGGCCTCCCACACAATCTT
	CaCZSOD4-Rev	TATTGGAGTCAAACCAACAAC

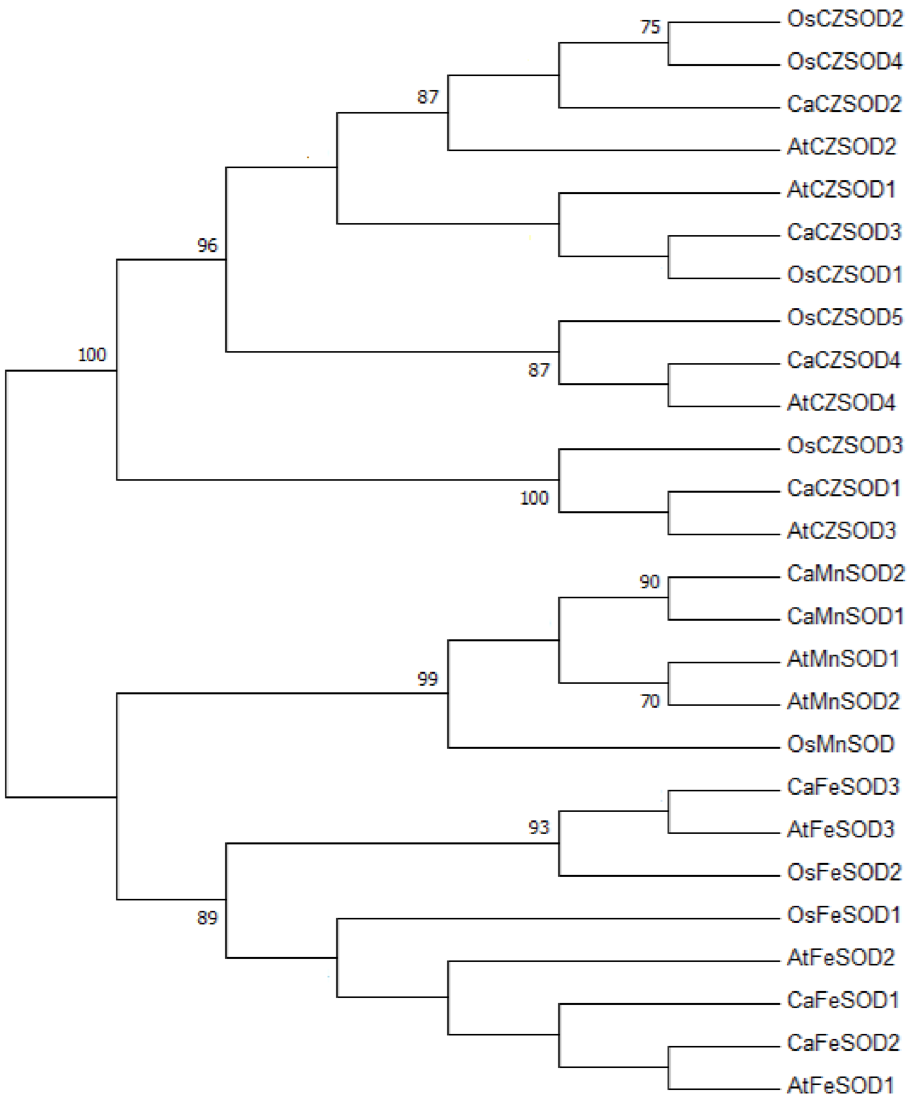

Figure S1. Phylogenetic tree of SOD proteins from *Arabidopsis thaliana*, rice (*Oryza sativa*), and chili pepper. The tree was constructed using the neighbor-joining method with 1,000 bootstrap replicates in MEGA 7.
